# Ti_3_C_2_T*_x_* Coated with TiO_2_ Nanosheets for the Simultaneous Detection of Ascorbic Acid, Dopamine and Uric Acid

**DOI:** 10.3390/molecules29122915

**Published:** 2024-06-19

**Authors:** Dengzhou Jia, Tao Yang, Kang Wang, Hongyang Wang, Enhui Wang, Kuo-Chih Chou, Xinmei Hou

**Affiliations:** 1 Institute for Carbon Neutrality, University of Science and Technology Beijing, Beijing 100083, China; 2 Institute of Steel Sustainable Technology, Liaoning Academy of Materials, Shenyang 110167, China; 3 State Key Laboratory of Environmental Criteria and Risk Assessment, Chinese Research Academy of Environmental Sciences, Beijing 100012, China; 4 Beijing Advanced Innovation Center for Materials Genome Engineering, University of Science and Technology Beijing, Beijing 100083, China

**Keywords:** dopamine, ascorbic acid, uric acid, Ti_3_C_2_T*_x_*, TiO_2_, solvothermal, electrochemical sensor

## Abstract

Two-dimensional MXenes have become an important material for electrochemical sensing of biomolecules due to their excellent electric properties, large surface area and hydrophilicity. However, the simultaneous detection of multiple biomolecules using MXene-based electrodes is still a challenge. Here, a simple solvothermal process was used to synthesis the Ti_3_C_2_T*_x_* coated with TiO_2_ nanosheets (Ti_3_C_2_T*_x_*@TiO_2_ NSs). The surface modification of TiO_2_ NSs on Ti_3_C_2_T*_x_* can effectively reduce the self-accumulation of Ti_3_C_2_T*_x_* and improve stability. Glassy carbon electrode was modified by Ti_3_C_2_T*_x_*@TiO_2_ NSs (Ti_3_C_2_T*_x_*@TiO_2_ NSs/GCE) and was able simultaneously to detect dopamine (DA), ascorbic acid (AA) and uric acid (UA). Under concentrations ranging from 200 to 1000 μM, 40 to 300 μM and 50 to 400 μM, the limit of detection (LOD) is 2.91 μM, 0.19 μM and 0.25 μM for AA, DA and UA, respectively. Furthermore, Ti_3_C_2_T*_x_*@TiO_2_ NSs/GCE demonstrated remarkable stability and reliable reproducibility for the detection of AA/DA/UA.

## 1. Introduction

MXene, a type of two-dimensional (2D) material, is characterized by the formula M_n+1_X_n_T. In this formula, M represents transition metals, X could be either carbon or nitrogen, while T refers to surface functional groups like =O, –F, and –OH [1,2], and it attracts attention as a promising material for electrochemical modification [3,4]. In particular, the abundant functional groups, high specific surface area and excellent electrical properties of MXene provide promising applications for the electrochemical detection of various biomolecules, especially for dopamine (DA), ascorbic acid (Vitamin C or AA) and uric acid (UA). The stability of physiological functions can be influenced by these three important substances which coexist in body fluids [5]. As a poly-hydroxyl molecule, AA plays a dual role in promoting antibody production and neutralizing the harmful impact of free radicals [6]. DA, a catecholamine neurotransmitter, contributes to nervous system regulation [7]. UA serves as a purine metabolite, commonly present in living organisms [8]. Several common diseases are attributed to fluctuations in the concentrations of these biomolecules, including mental illness, AIDS, Parkinson’s disease, skin rashes and gout [9,10]. Thus, it is important to discover an appropriate approach for swiftly and precisely identifying AA, DA and UA.

Recently, the use of MXene as a modified electrode material has been shown to enable accurate detection of AA, DA and UA [11,12]. However, two factors limit the application of MXene: On the one hand, the unstable multilayer structure of MXene is prone to self-accumulation, which reduces the active sites and lowers the electron transfer rate [13]. On the other hand, the functional groups on the surface of MXene caused by wet etching are unable to form covalent bonds with the biomolecules, thus reducing the detection sensitivity [14]. Therefore, several strategies have been developed to resolve the above problems, such as the use of intercalating agents and surface modification/functionalization [15,16]. Notably, the preparation of composites (or heterojunctions) by introducing other active materials into MXenes is considered to be the best strategy. For instance, a novel MXene/DNA/Pd/Pt material was synthesized by Zheng et al., and the sensor exhibited high selectivity against AA and H_2_O_2_ [17]. Amara et al. fabricated a graphitic pencil which is modified by perylene diimide/MXene (Ti_3_C_2_T*_x_*), realizing the specific detection of DA [18]. However, the performance of MXene-based materials in simultaneously detecting AA/DA/UA remains unsatisfactory due to the significant concentration differences among different biomolecules [19], their similar oxidation potentials [20] and the strong electrostatic repulsion [21]. TiO_2_ is recognized for its considerable potential for use in the biological sector, thanks to its affordability, customizable attributes, lack of toxicity, simple preparation process, consistent quality and improved durability [22,23].

Furthermore, the nanocomposite of MXene@TiO_2_ prevents the aggregation of MXene, and it improves the stability of the electrochemical sensor and achieves reliable reproducibility [24]. So far, different forms of TiO_2_ have been synthesized, including nanoparticles, thin films structures, nanorods and so on [25,26]. In our previous work [27], it was mentioned that materials that have a high specific surface area can enable the precise detection of AA/DA/UA because of the presence of numerous active sites. Therefore, the growth of TiO_2_ nanosheets on the MXene surface can be used as a means to effectively realize the simultaneous detection of biological small molecules.

Here, Ti_3_C_2_T*_x_* coated with TiO_2_ nanosheets (Ti_3_C_2_T*_x_*@TiO_2_ NSs) is synthesized through a simple solvothermal process. By incorporating TiO_2_ nanosheets, Ti_3_C_2_T*_x_*@TiO_2_ NSs, modified glassy carbon electrode (Ti_3_C_2_T*_x_*@TiO_2_ NSs/GCE) enables detection of AA/DA/UA individually and simultaneously. Additionally, Ti_3_C_2_T*_x_*@TiO_2_ NSs/GCE demonstrates outstanding stability and reliable reproducibility.

## 2. Result and Discussion

### 2.1. Microstructural Characterizations of Ti_3_C_2_T_x_@TiO_2_ NSs Samples

As shown in Figure S1, comparing the standard cards (JCPDS Card No. 52-0875), it can be seen that the black curves show peaks at 19.1°, 34°, 36.77°, 38.94°, 41.7°, 48.3° and 60.14° corresponding to the (004), (101), (103), (104), (105), (107) and (110) crystalline facet characteristic peaks of Ti_3_AlC_2_. The red curve shows the peaks at 9.0°, 18.6°, 27.9° and 61.1°, which correspond to the (002), (006), (008) and (110) facets of Ti_3_C_2_T*_x_* [28]. Notably, the characteristic peak at 39.3° disappears, indicating the etching of the Al element [29]. Upon etching of the Al element, TiO_2_ grows on the MXene surface due to oxidation, where peaks at 36.9°, 37.8° and 38.6° (corresponding to the (103), (004) and (112) crystal planes of TiO_2_) align with characteristic peaks of the (103) crystal planes of Ti_3_C_2_T*_x_*, leading to widened peaks at 35°–40°. Prior to annealing (blue curve), the shift of the (002) peak toward lower angles suggests further delamination of Ti_3_C_2_T*_x_*. Additionally, comparing the standard cards (JCPDS card NO. 21-1272), two faint peaks at 38.6° and 44.1° emerge, corresponding to the (112) and (200) planes of anatase TiO_2_. Following annealing (green curve), another peak appears at position 23.5°, corresponding to the (101) planes of anatase TiO_2_. After solvothermal treatment, the results show that the lamellae structure of Ti_3_C_2_T*_x_* remains stable, while a crystallized phase of anatase TiO_2_ has emerged.

**Figure 1:**
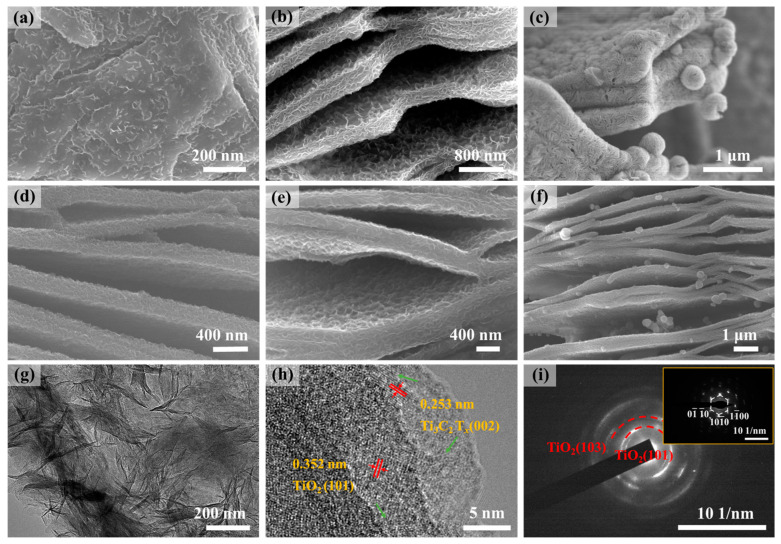
SEM images of Ti_3_C_2_T*_x_*@TiO_2_ NSs(0.6): (**a**) Ti_3_C_2_T*_x_*@TiO_2_ NSs(0.8), (**b**) Ti_3_C_2_T*_x_*@TiO_2_ NSs(1.0), (**c**) SEM images of Ti_3_C_2_T*_x_*@TiO_2_ NSs(0.6), (**d**) Ti_3_C_2_T*_x_*@TiO_2_ NSs(0.8), (**e**) Ti_3_C_2_T*_x_*@TiO_2_ NSs(1.0), (**f**) after annealed at 350 °C, (**g**) TEM image, (**h**) HR-TEM and (**i**) SAED image of Ti_3_C_2_T*_x_*@TiO_2_ NSs(0.8).

Figure S2a depicts the closely aligned layered structure of the pristine Ti_3_AlC_2_. As shown in Figure S2b, the Ti_3_C_2_T*_x_* exhibits a layered accordion-like structure following treatment with HF (24 h), with each lamella measuring around 20 nm thick and a layer spacing of approximately 300 nm. Figure 1a–c illustrate the comparison of samples with varying amounts of TiCl_3_. When the TiCl_3_ concentration is low (Ti_3_C_2_T*_x_*@TiO_2_ NSs(0.6), Figure 1a), sparse NSs can be observed on the surface of Ti_3_C_2_T*_x_* lamellae. As the amount of TiCl_3_ increases (Ti_3_C_2_T*_x_*@TiO_2_ NSs(0.8), Figure 1b), the density of TiO_2_ NSs on the surface of Ti_3_C_2_T*_x_* lamellae gradually increases. At the maximum TiCl_3_ concentration (Ti_3_C_2_T*_x_*@TiO_2_ NSs(1.0), Figure 1c), highly dense NSs, and even nanospheres composed of NSs, are formed on the surface of Ti_3_C_2_T*_x_* lamellae, indicating an excess of TiCl_3_. After annealing, the morphology remains relatively unchanged (Figure 1d–f). The TEM images of Ti_3_C_2_T*_x_*@TiO_2_ NSs(0.8) are presented in Figure 1g,h. Figure 1g shows abundant NSs growing on the surface of Ti_3_C_2_T*_x_* lamellae, with lengths of 30–100 nm and thicknesses of 3–5 nm. A facet spacing of 0.253 nm wide can be observed in the edge portion of the material, corresponding to the (002) crystallographic facet of Ti_3_C_2_T*_x_*. The lattice stripe spacing (0.352 nm) of the NSs is correlated to the (101) crystal plane of anatase TiO_2_ [30] (Figure 1h). The SAED image of Ti_3_C_2_T*_x_*@TiO_2_ NSs is demonstrated in Figure 1i (red). The diffraction circle diameters in the SAED plots are the same as those diffracted from the (101) and (103) crystal surfaces of anatase TiO_2_, while the six bright and sharp spots exhibit a six-fold symmetry, representing the crystal planes (0$\bar{1}\bar{1}$0), (10$\bar{1}$0) and ($1\bar{1}$00) of Ti_3_C_2_T*_x_* (Figure 1i, inset).

**Figure 2:**
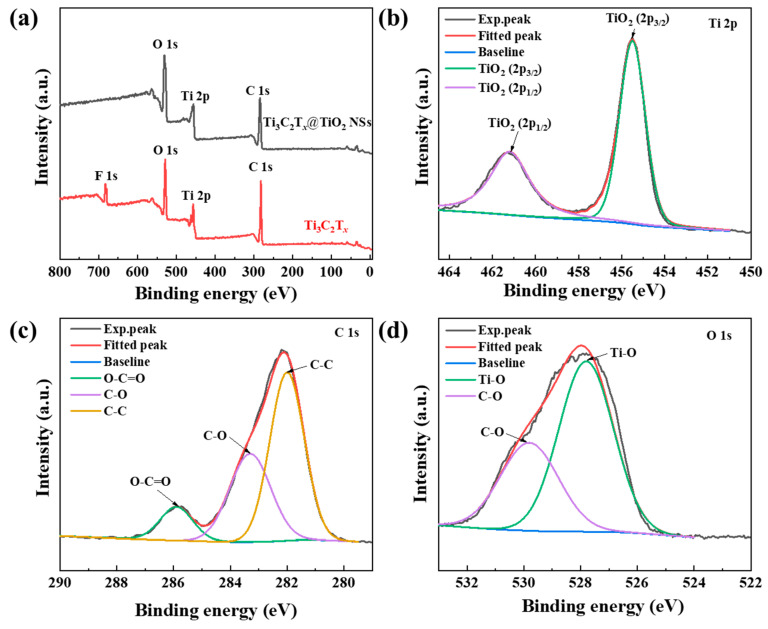
XPS spectra of the (**a**) survey, (**b**–**d**) Ti 2p, C 1s and O 1s for the Ti_3_C_2_T*_x_*@TiO_2_ NSs.

Figure 2a shows the full XPS spectra of Ti_3_C_2_T*_x_* and Ti_3_C_2_T*_x_*@TiO_2_ NSs. The red curve in Figure 2a represents Ti_3_C_2_T*_x_*, exhibiting characteristic peaks for C 1s, Ti 2p, O 1s and F 1s at 282 eV, 457 eV, 529 eV and 683 eV, respectively. The black curve in Figure 2a corresponds to Ti_3_C_2_T*_x_*@TiO_2_ NSs, where the characteristic peak at 682 eV for F 1s is absent. This observation suggests that the Ti^3+^ of TiCl_3_ has replaced the –F functional groups. The Ti 2p profile (Figure 2b) reveals the energy levels of 461.2 and 457.2 eV, corresponding to Ti 2p_1/2_ and Ti 2p_3/2_, which indicate the formation of anatase TiO_2_. Concerning the C 1s profile (Figure 2c), characteristic peaks occur at 285.8, 283.2 and 281.7 eV, corresponding to O-C=O, C–O, and C–C, respectively. Furthermore, the O 1s profile (Figure 2d) shows peaks at 529.5 and 527.3 eV that represent C–O and Ti–O bonds in TiO_2_, respectively. These findings provide evidence for the disappearance of –F functional groups and the concomitant generation of TiO_2_ on the surface of Ti_3_C_2_T*_x_*.

**Figure 3:**
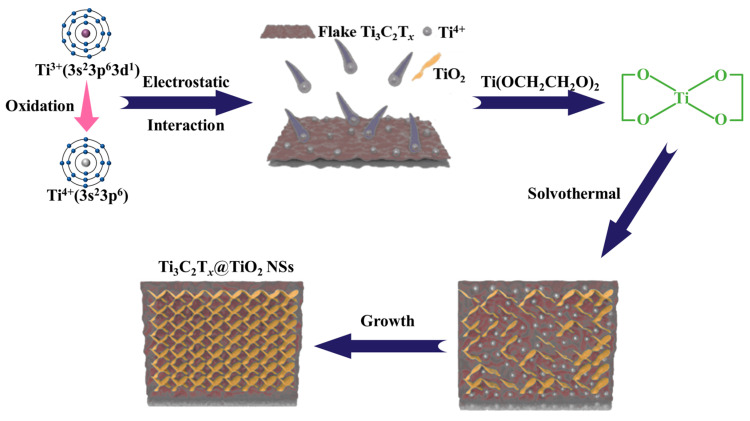
Growth mechanism of TiO_2_ nanosheet on Ti_3_C_2_T*_x_* surface.

The process of morphological evolution involved in the fabrication of Ti_3_C_2_T*_x_* @TiO_2_ NSs is illustrated in Figure 3. Initially, Ti^4+^ ions are generated through the reaction of Ti^3+^ of TiCl_3_ with dissolved oxygen in the solution [31,32] (Equations (S1) and (S2)). Subsequently, these positively charged Ti^4+^ ions uniformly adhere to the negatively charged surface of Ti_3_C_2_T*_x_*. Concurrently, Ti^4+^ ions react with ethylene glycol to form titanium glycolate crystal nucleuses (Ti(OCH_2_CH_2_O)_2_) [33] (Equation (S3)). As the solvothermal process proceeds, a portion of Ti(OCH_2_CH_2_O)_2_ undergoes hydrolysis, leading to the development of a small quantity of anatase TiO_2_ on the surface of Ti_3_C_2_T*_x_*. In the final annealing step, the remaining unreacted Ti(OCH_2_CH_2_O)_2_ is completely transformed into anatase TiO_2_, while the carbon-containing species are entirely eliminated (Equation (S4)).

### 2.2. Electrochemical Behaviors of Ti_3_C_2_T_x_@TiO_2_ NSs

**Figure 4:**
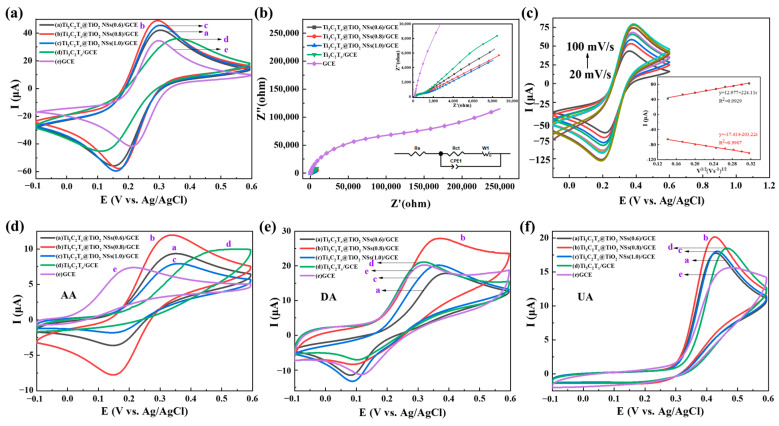
(**a**) CV curves and (**b**) EIS curve of Ti_3_C_2_T*_x_*@TiO_2_ NSs/GCE, Ti_3_C_2_T*_x_*/GCE and GCE; (**c**) CV curves of Ti_3_C_2_T*_x_*@TiO_2_ NSs(0.8)/GCE with different scanning rates. The CV curves of Ti_3_C_2_T*_x_*@TiO_2_ NSs/GCE, Ti_3_C_2_T*_x_*/GCE and GCE are detected in (**d**) 100 μMAA, (**e**) 100 μM DA and (**f**) 100 μM UA solutions. In all the experiments, detection was in 0.1 M KCl solution with 0.5 mM K_3_[Fe (CN)_6_].

Figure 4a shows the electrochemical performance of Ti_3_C_2_T*_x_*@TiO_2_ NSs/GCE, Ti_3_C_2_T*_x_*/GCE and GCE using CV curves in 0.1 M KCl solution with 0.5 mM K_3_[Fe (CN)_6_]. The relative peak currents, listed in decreasing order, are as follows: Ti_3_C_2_T*_x_*@TiO_2_ NSs(0.8)/GCE > Ti_3_C_2_T*_x_*@TiO_2_ NSs(1.0)/GCE > Ti_3_C_2_T*_x_*@TiO_2_ NSs(0.6)/GCE > Ti_3_C_2_T*_x_*/GCE > GCE. As shown in Figure 4b, the impedance of the different electrodes was obtained by EIS (voltage amplitude of 10 mV, 10^−2^–10^6^ Hz). The electron transfer process occurring at the electrode surface was indicated by the semicircular arc observed at higher frequencies. The charge transfer resistance (*R*_ct_) of the electrodes can be represented by the semicircular arc diameter of the fitted plot line. Based on the Randles equivalent circuit (Figure 4b, inset), Ti_3_C_2_T*_x_*@TiO_2_ NSs(0.8)/GCE exhibits the lowest charge transfer impedance (*R*_ct_ = 531 Ω), followed by Ti_3_C_2_T*_x_*/GCE (868.7 Ω), Ti_3_C_2_T*_x_*@TiO_2_ NSs(0.6)/GCE (608.7 Ω) and Ti_3_C_2_T*_x_*@TiO_2_ NSs(1.0)/GCE (1183 Ω).

As shown in Figure 4c, which displays the CV curves, the peak current of Ti_3_C_2_T*_x_*@TiO_2_ NSs(0.8)/GCE rises in a linear fashion as the scan rate increases (20–100 mV/s). Furthermore, the redox peaks of the electrodes display a good linear relationship with the square root of scan rate (the inset of Figure 4c). The active surface area of Ti_3_C_2_T*_x_*@TiO_2_ NSs/GCE was calculated using the Randles–Sevcik equation:

(1) $$Ip=2.69\times 10^{5}\times n^{3/2}\times AC^{*}\times D^{1/2}\times {ʋ}^{1/2}$$

where *I_p_* represents the peak oxidation current, *n* is the number of electron transfers (*n* = 1), *A* is the active surface area, *D* is the diffusion coefficient (7.6 × 10^−6^ cm^2^ s^−1^ for [Fe(CN)_6_]^4+^), C* is the concentration of [Fe(CN)_6_]^4+^ and ʋ is the scanning rate. The active surface area of Ti_3_C_2_T*_x_*@TiO_2_ NSs(0.8)/GCE was calculated as approximately 0.74 cm^2^, which is nine times greater than that of bare GCE (0.08 cm^2^). The TiO_2_ NSs grown on the surface of Ti_3_C_2_T*_x_* effectively increased the area of catalytic activity. As shown in Figure 4d, the reaction peak could not be detected by Ti_3_C_2_T*_x_*/GCE. The oxidation peak of AA is shifted to a lower reaction potential when Ti_3_C_2_T*_x_*@TiO_2_ NSs/GCE is employed. The peak current, listed in descending order, is as follows: Ti_3_C_2_T*_x_*@TiO_2_ NSs(0.8)/GCE > Ti_3_C_2_T*_x_*@TiO_2_ NSs(0.6)/GCE > Ti_3_C_2_T*_x_*@TiO_2_ NSs(1.0)/GCE > GCE. As shown in Figure 4e, all five electrodes can detect the oxidation peak of DA, with the peak current ordered as follows: Ti_3_C_2_T*_x_*@TiO_2_ NSs(0.8)/GCE > Ti_3_C_2_T*_x_*/GCE > GCE > Ti_3_C_2_T*_x_*@TiO_2_ NSs(1.0)/GCE > Ti_3_C_2_T*_x_*@TiO_2_ NSs(0.6)/GCE. The five oxidation peaks of UA are clearly distinguished, with the peak current ordered as follows: Ti_3_C_2_T*_x_*@TiO_2_ NSs(0.8)/GCE > Ti_3_C_2_T*_x_*/GCE > Ti_3_C_2_T*_x_*@TiO_2_ NSs(1.0)/GCE > Ti_3_C_2_T*_x_*@TiO_2_ NSs(0.6)/GCE > GCE (Figure 4f). Since Ti_3_C_2_T*_x_*@TiO_2_ NSs(0.8)/GCE showed excellent electrochemical detection capability, it was used as the modified electrode material for the additional experiments.

**Figure 5:**
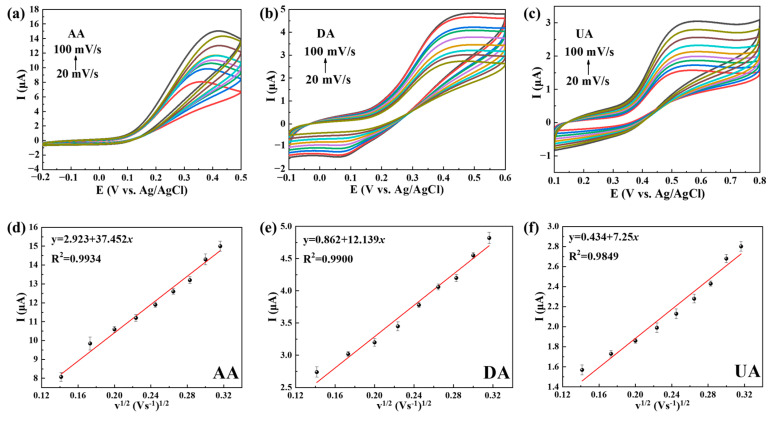
CV curves recorded in (**a**) 100 μM AA, (**b**) 100 μM DA and (**c**) 100 μM UA, with increasing scan rates on Ti_3_C_2_T*_x_*@TiO_2_ NSs/GCE. (**d**–**f**) The square root of the scan rates vs. peak current of AA/DA/UA. Error bars based on *S/N* = 3.

The oxidation kinetics of AA, DA and UA on the Ti_3_C_2_T*_x_*@TiO_2_ NSs/GCE electrode were studied using CV (pH 7.4) under various scan rates (20 mV/s–100 mV/s). The peak currents of AA, DA and UA show strong linear correlation with the scan rate (Figure 5a–c), while the peak potentials are gradually biased toward large overpotentials, indicating kinetic limitations in the reaction [34]. As shown in Figure 5d–f, the linear correlation coefficient (R^2^) between the oxidation current and square root of the scan rate was determined to be 0.9868, 0.9801 and 0.9700 for AA, DA and UA. Hence, it can be confirmed that the oxidation reaction of AA, DA and UA on Ti_3_C_2_T*_x_*@TiO_2_ NSs/GCE was controlled by a diffusion process [35].

### 2.3. The Use of Ti_3_C_2_T_x_@TiO_2_ NSs/GCE for Separate and Simultaneous Detection of AA, DA and UA

**Figure 6:**
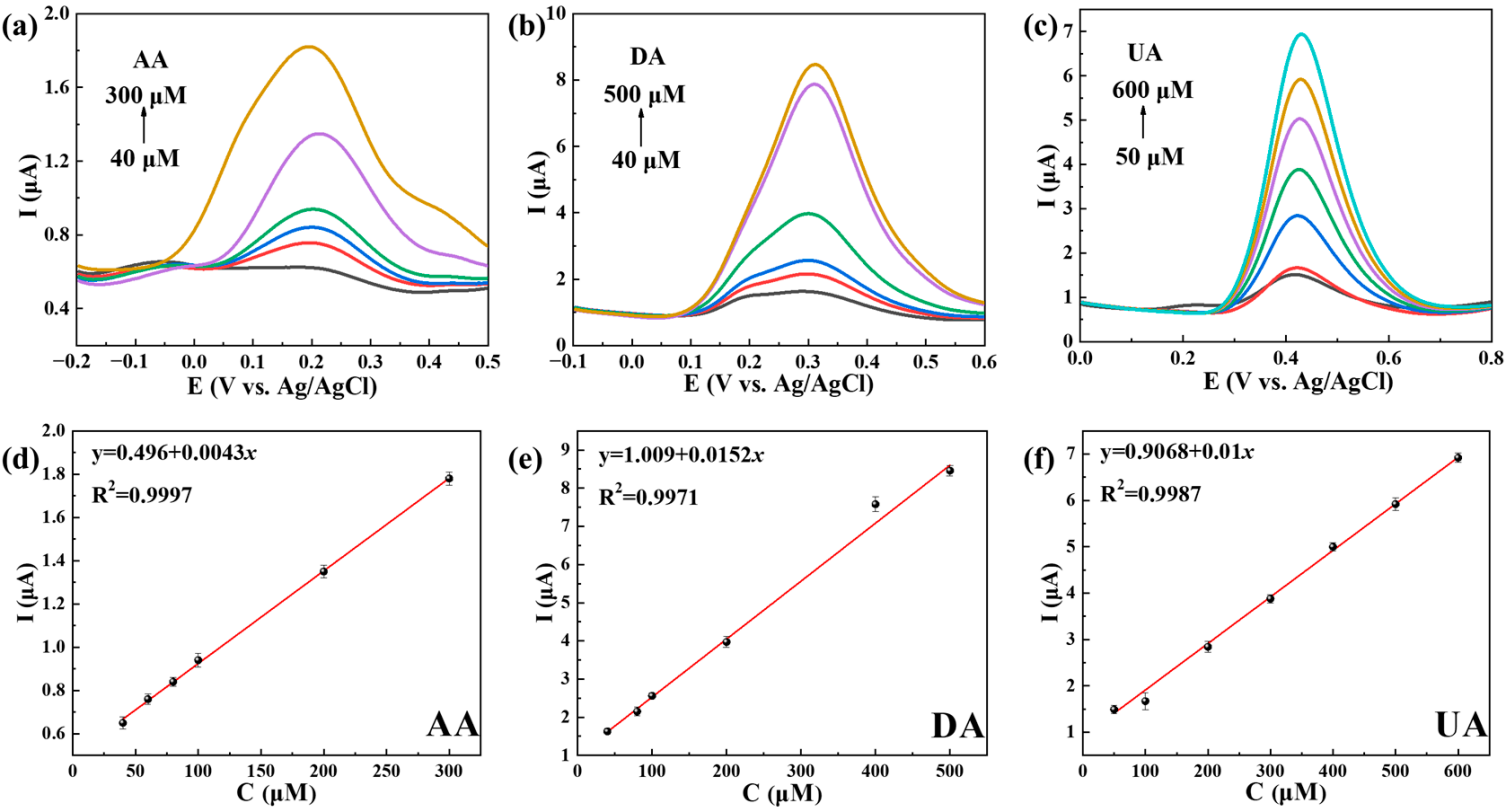
DPV curves of (**a**) AA, (**b**) DA and (**c**) UA, separately detected on Ti_3_C_2_T*_x_*@TiO_2_ NSs/GCE, and the corresponding oxidation concentration vs. peak current. (**d**–**f**). Error bars based on *S/N* = 3.

Figure 6 demonstrates the separate detection of AA, DA and UA on Ti_3_C_2_T*_x_*@TiO_2_ NSs/GCE in 0.1 M PBS (pH 7.4) through the use of DPV. Figure 6a–c show thatTi_3_C_2_T*_x_*@TiO_2_ NSs/GCE exhibits the individual detection of AA, DA and UA under concentrations ranging from 40 to 300 μM, 40 to 500 μM and 50 to 600 μM, respectively. Figure 6d–f show the R^2^a of AA, DA and UA as 0.9994, 0.9941 and 0.9975, respectively. The LOD for AA, DA and UA was determined to be 1.12 μM, 0.35 μM and 0.47 μM, respectively.

**Figure 7:**
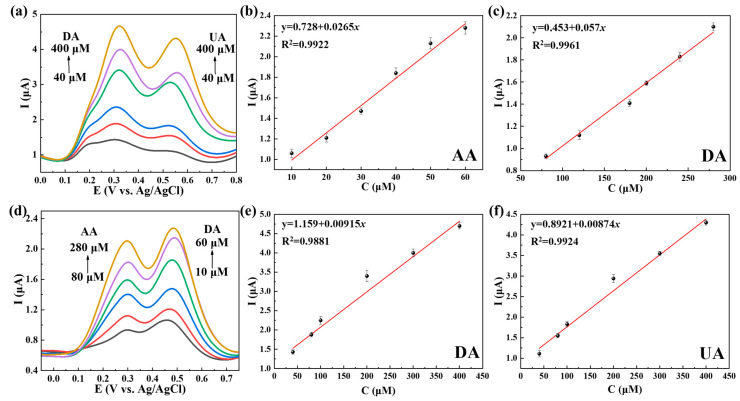
DPV curves based on different concentrations when detecting (**a**) AA and DA and (**d**) DA and UA on Ti_3_C_2_T*_x_*@TiO_2_ NSs/GCE in 0.1 M PBS (pH 7.4). (**b**,**c**) Calibration curves for the current vs. concentrations of AA and UA, (**e**,**f**) calibration curves for the current vs. concentrations of DA and UA. Error bars based on *S/N* = 3.

Figure 7a demonstrates the simultaneous determination of AA and DA through DPV on Ti_3_C_2_T*_x_*@TiO_2_ NSs/GCE. A noticeable difference was observed in the peak potentials of AA (0.27 V) and DA (0.46 V). For AA (80–280 μM) and DA (10–60 μM), a direct correlation was identified between peak currents and concentrations, resulting in R^2^ values of 0.9845 and 0.9922, respectively (Figure 7b,c). Similarly, Figure 7d illustrates the distinct separation of DA (0.33 V) and UA (0.54 V) oxidation peak potentials. As shown in Figure 7e,f, a linear response was observed for DA (40–400 μM) and UA (40–400 μM) with R^2^ values of 0.9764 and 0.9841, respectively. Meanwhile, it can be observed that altering the concentration of a single solute resulted in a corresponding linear change in the detection current of the AA–UA (Figures S3 and S4). These results serve as evidence for the dependable detection sensitivity exhibited by Ti_3_C_2_T*_x_*@TiO_2_ NSs/GCE.

It should be noted that when AA and UA coexisted in solution, no discernible peak splitting phenomenon was observed (Figure S5a–c). At the same time, the oxidation potential was shifted higher upon the addition of AA or UA. This phenomenon is attributed to the close oxidation potential of AA and UA; the offset of the AA and UA peaks is due to the catalytic oxidation of UA by AA [36].

**Figure 8:**
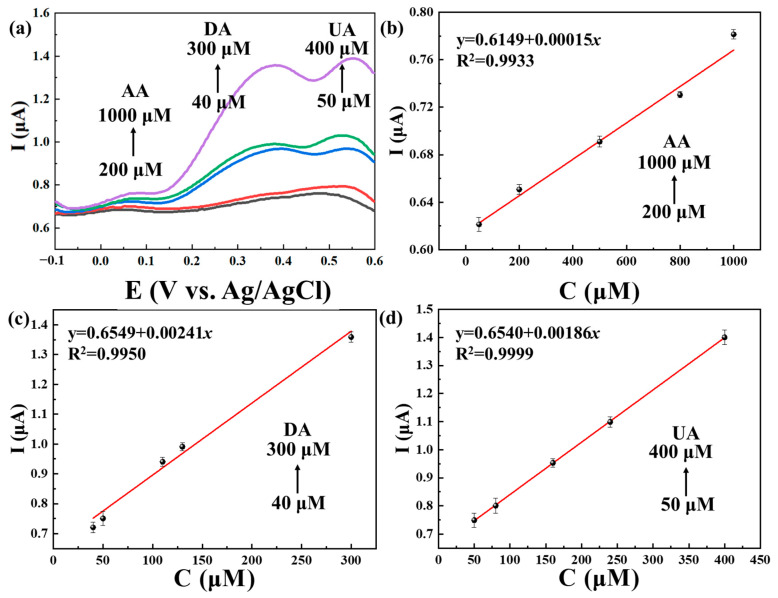
(**a**) DPVs recorded for Ti_3_C_2_T*_x_*@TiO_2_ NSs/GCE for different concentrations of AA (200–1000 μM), DA (40–300μM) and UA (50–400μM). (**b**–**d**) Linear calibration curves for the oxidation peak currents vs. concentrations of AA, DA and UA. Error bars based on *S/N* = 3.

The DPV curves of the simultaneous determination of AA, DA and UA using Ti_3_C_2_T*_x_*@TiO_2_ NSs/GCE are shown in Figure 8. As depicted in Figure 8a, the oxidation peak potentials of AA, DA and UA were observed to be 0.06 V, 0.37 V and 0.55 V, respectively. Additionally, Figure 8b–d highlight the linear relationship between peak currents and concentrations, with observed ranges of 200–1000 μM (R^2^ = 0.9875) for AA, 40–300 μM (R^2^ = 0.9875) for DA and 50–400 μM (R^2^ = 0.9991) for UA. The LOD was determined to be 2.91 μM, 0.19 μM and 0.25 μM for AA, DA and UA, respectively. Subsequently, this work was compared with previous work on electrochemical sensors (Table 1). There are several key advantages in our work: Firstly, our synthesis method is simple, and the preparation conditions are gentle, in contrast to the complex synthesis processes and high-temperature preparation conditions commonly found in the existing literature [37,38,39]. Secondly, our use of non-precious metals is more cost-effective than the use of precious metals; even though precious metals can enhance material sensitivity, they also escalate manufacturing costs [40,41]. Materials such as MXene and TiO_2_ present a promising low-cost alternative. Lastly, our work demonstrates a lower Limit of Detection (LOD) for AA when using MXene-based materials. While the negative electronegativity of the MXene surface traditionally hinders AA detection, the TiO_2_ synthesized via the solvothermal method effectively optimizes the surface charge of MXene, enabling the simultaneous detection of AA, DA and UA, with a particularly noteworthy LOD of 2.9 μM for AA.

It is noteworthy that the inclusion of DA exhibited an ability to separate the overlapping peaks of AA and UA. The oxidation products of AA and UA absorb or electropolymerize on the electrodes and reduce their detection sensitivity [42,43]. When DA is added, the oxidation products of DA react with AA, thereby mitigating electrode contamination. Additionally, DA is recognized as a catalyst for both AA and UA [44,45]. Upon the addition of DA, it underwent reactions with AA and UA to generate intermediate complexation products, effectively lowering the activation energy of AA and increasing the activation energy of UA. Consequently, the peaks corresponding to AA and UA resurfaced during simultaneous detection.

**Table 1:** The performance of sensors for the electrochemical simultaneous detection of AA, DA and UA has been reported recently.

Electrode	Linear Range (μM)			LOD (μM)			Refs.
	AA	DA	UA	AA	DA	UA	
FGP	10–1800	1–300	5–800	4.2	0.42	2.2	[46]
P-4-ABA/GCE	20–800	5–100	1–80	5.0	1.0	0.5	[47]
HNP-PtTi	200–1000	4–500	100–1000	24.2	3.2	5.3	[48]
p-TA/nano-Au/GCE	2.1–50.1	0.6–340	1.6–110	14.8	0.05	0.1	[49]
Fe_3_O_4_/Co_3_O_4_/mc@g-C_3_N_4_/GCE	500–8000	1–70	5–100	12.55	0.21	0.18	[50]
SPGNE	4–4500	0.5–2000	0.8–2500	0.95	0.12	0.20	[8]
PG/GCE	9–2314	5–710	6–1330	6.45	2.00	4.82	[51]
Pt@g-C_3_N_4_/N-CNTs/GCE	2–2000	5–100	1–110	29.44	0.21	2.99	[52]
Co/MoSe_2_/PPy@CNF	30–3212	1.2–536	10–1071	6.32	0.45	0.81	[37]
Ti_3_C_2_T*_x_*/TiO_2_ NSs	200–1000	40–300	50–400	2.9	0.19	0.25	This work

FGP—Few-layer graphene/Pt; GCE—glassy carbon electrode; HNP—hierarchical nanoporous; P-4-ABA—Poly (4-aminobutyric acid); p-TA/nano-Au—gold nanoparticles on poly (3-amino-5-mercapto-1,2,4-triazole) film; Fe_3_O_4_—iron oxide; Co_3_O_4_—cobalt oxide; SPGNE—screen-printed graphene electrode; g-C_3_N_4_—graphitic carbon nitride; N-CNTs—N-doped carbon nanotubes; PG—pristine graphene; and Co/MoSe_2_/PPy@CNF—cobalt-doped 2D-MoSe_2_/polypyrrole hybrid-based carbon nanofibers.

Based on previous studies [40,41], simultaneous detection of AA/DA/UA faces significant challenges due to mutual interference, such as strong electrostatic interactions and overlapping oxidation potentials [36,38,53]. Through the solvothermal process, Ti_3_C_2_T*_x_*@TiO_2_ NSs exhibit two distinct advantages that enhance their ability to detect these molecules simultaneously. First, the replacement of –F by additional Ti^3+^ ions significantly changes the surface charge of Ti_3_C_2_T*_x_*, reducing the mutual repulsion of the analytes. Secondly, a considerable number of TiO_2_ NSs are grown on the Ti_3_C_2_T*_x_* lamellae surface, which decreases the ion diffusion length [54] and increases the specific surface area of the Ti_3_C_2_T*_x_*@TiO_2_ NSs. Finally, the formation of Ti_3_C_2_T*_x_* and TiO_2_ heterojunctions facilitates the transport of charge carriers [39]. As a result, the integration of Ti_3_C_2_T*_x_* and TiO_2_ allows for the creation of a highly precise and sensitive sensor capable of detecting biological small molecules with high resolution.

### 2.4. Reproducibility, Interference Immunity Testing and Stability Analysis

**Figure 9:**
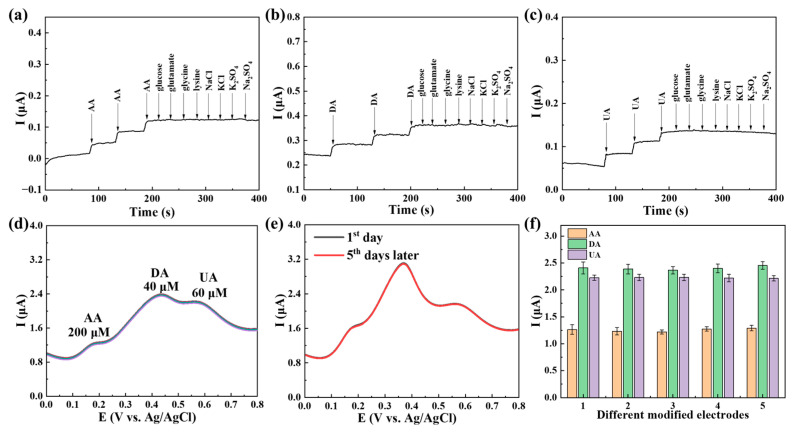
Amperometric responses of the mixture upon addition of 10 μM (**a**) AA, (**b**) DA, (**c**) UA and 1000 μM other chemicals on Ti_3_C_2_T*_x_*@TiO_2_ NSs/GCE; (**d**) DPV curves detected 5 consecutive times on Ti_3_C_2_T*_x_*@TiO_2_ NSs/GCE for AA, DA and UA; (**e**) DPV curve change of Ti_3_C_2_T*_x_*@TiO_2_ NSs/GCE containing 200 μM AA, 40 μM DA and 60 μM UA (5 days) in 0.1 M PBS; (**f**) peak current using five different Ti_3_C_2_T*_x_*@TiO_2_ NSs/GCE electrodes at fixed concentrations of AA (200 μM), DA (40 μM) and UA (60 μM) in PBS. Error bars based on *S/N* = 3.

Several potential coexisting substances were detected using the amperometric method to verify the interference resistance of Ti_3_C_2_T*_x_*@TiO_2_ NSs/GCE (Figure 9a–c). The continuous addition of the interfering substance did not affect the detection current of the AA/DA/UA. Therefore, Ti_3_C_2_T*_x_*@TiO_2_ NSs/GCE demonstrated excellent anti-interference capability for detecting AA, DA and UA. Five consecutive measurements were conducted in the solution containing 200 μM AA, 60 μM DA and 40 μM UA, investigating the stability of Ti_3_C_2_T*_x_*@TiO_2_ NSs/GCE. The results shown in Figure 9d indicate that the peaks of the five consecutive measurements remained almost unchanged, with a calculated RSD of 0.54%. This suggests that Ti_3_C_2_T*_x_*@TiO_2_ NSs/GCE exhibits good stability under testing conditions. Furthermore, a DPV test was performed after the electrode was exposed to air for 5 days to evaluate the environmental stability of Ti_3_C_2_T*_x_*@TiO_2_ NSs/GCE (Figure 9e). The peak currents remained nearly constant, indicating the excellent environmental stability of Ti_3_C_2_T*_x_*@TiO_2_ NSs/GCE. As shown in Figure 9f, the simultaneous detection of AA/DA/UA was utilized on five different Ti_3_C_2_T*_x_*@TiO_2_ NSs/GCE electrodes in 0.1 M PBS using DPV. The oxidation peak currents of AA (200 M), DA (40 M) and UA (60 M) were not significantly altered.

## 3. Experimental Section

### 3.1. Chemicals

Kaixi Materials Co., Ltd. In Shandong, China supplied Ti_3_AlC_2_ powder (94 wt%, ~300 mesh). Sinopharm Chemical Reagent Co., Ltd. in Beijing, China provided potassium chloride (KCl), ethyl alcohol (99.9%), titanium trichloride (TiCl_3_), hydrofluoric acid (40 wt% HF), acetone, potassium ferricyanide (K_3_[Fe (CN)_6_]), ethylene glycol (EG), ascorbic acid, dopamine, uric acid, dibasic sodium phosphate (Na_2_HPO_4_) and potassium dihydrogen phosphate (KH_2_PO_4_). Praxair Gas Co., Ltd. supplied nitrogen (N_2_) and argon gas (Ar). Yue Ci Electronic Technology Co., Ltd. in Shanghai, China provided aluminum oxide powder (1.0, 0.3 and 0.05 μm). Analytical grade chemicals were employed in this work without additional purification.

### 3.2. Apparatus

An electrochemical workstation was used to obtain the electrochemical properties of the modified electrode. The model of the electrochemical workstation is CHI, 660E (Dalian, China). The surface morphology of the material was obtained by scanning electron microscopy (SEM) using the Nova 230, FEI (Hillsboro, OR, USA) model. The ultrastructure of the material was obtained by transmission electron microscopy, model Tecnai G2 F30 S-TWIN, FEI (USA). The high-resolution electron microscopy was obtained using JEM-2010 (HRTEM, JEOL Japan Electronics Co., Ltd, Akishima-shi in Japan). The structure and phase of the materials were analyzed using X-ray powder diffraction (XRD), model TTRIII, Rigaku (Tokyo, Japan) with Cu Kα radiation (λ = 1.5406 Å) and X-ray photoelectron spectroscopy (XPS), using the VG Multilab 2009 (Manchester, UK) model.

### 3.3. Synthesis of Ti_3_C_2_T_x_@TiO_2_ NSs

**Scheme 1:**
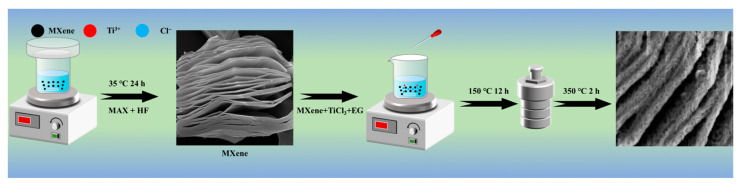
The synthesis process of Ti_3_C_2_T*_x_*@TiO_2_ NSs.

Scheme 1 shows the preparation process of Ti_3_C_2_T*_x_*@TiO_2_ NSs. Ti_3_C_2_T*_x_* was obtained by the one-step wet etching method [55]. The Ti_3_AlC_2_ powder (1 g) was added gradually to a solution of HF (40 mL, 40 wt%) and allowed to react at 35 °C for 24 h with stirring at a speed of 500 rpm. The etched substance was rinsed with deionized water (DI) until the pH reached above 6.0. High-speed centrifugation (300 rpm, 3 min) allows the powder to be separated from the DI. Finally, the gelatinous Ti_3_C_2_T*_x_* was dehydrated under vacuum freezing conditions (−80 °C, 2 h). A solution was prepared by mixing 30 mL of EG with an equal volume (*k* mL) of TiCl_3_ and DI (*k* = 0.6, 0.8, 1.0) and stirring it for approximately 30 min. Subsequently, 20 mg of as-prepared Ti_3_C_2_T*_x_* was added to the solution and sonicated for 30 min to create a homogeneous suspension. The suspension was moved to a stainless steel autoclave and subjected to heat treatment at 150 °C for 12 h before being cooled to room temperature. The precipitates were washed with ethyl alcohol, and dried for 2 days. Finally, the precipitates were annealed at 350 °C in flowing Ar for 2 h. The obtained granular powders were marked as Ti_3_C_2_T*_x_*@TiO_2_ NSs(0.6), Ti_3_C_2_T*_x_*@TiO_2_ NSs(0.8), and Ti_3_C_2_T*_x_*@TiO_2_ NSs(1.0), respectively.

### 3.4. Fabrication of the Ti_3_C_2_T_x_@TiO_2_ NSs Electrode

Aluminum oxide powder with particle sizes of 1.0, 0.3 and 0.05 μm was first placed on a nylon cloth for polishing the glassy carbon electrode (GCE). The electrodes were then washed continuously for 5 min using acetone, ethanol and deionized water (DI), respectively, until it was ensured that a smooth electrode surface was obtained. Then, 10 mg of Ti_3_C_2_T*_x_*@TiO_2_ NSs was dispersed in 10 mL of DI using the ultrasonic method. Next, 3 μL of the aforementioned Ti_3_C_2_T*_x_*@TiO_2_ NSs solution was dropped on the GCE surface and allowed to dry under nitrogen atmosphere to form a flat film. For comparison, the preparation process of the remaining electrodes was performed in a similar way.

### 3.5. Electrochemical Measurement

The main purpose of this work is to discuss the electrochemical detection performance of modified electrodes for AA/DA/UA in human physiological situations. Therefore, pH 7.4 was chosen as the detection solution condition in this work. The pH of the solution was regulated by PBS. GCE or Ti_3_C_2_T*_x_*/GCE or Ti_3_C_2_T*_x_*@TiO_2_ NSs/GCE served as the working electrode. Ag/AgCl was used as the reference electrode. Platinum (Pt) served as the counter electrode. The electrochemical detection capability was evaluated using differential pulse voltammetry (DPV). Furthermore, the time-amperometric method was used to assess the ability to resist interferences. The interferents included NaCl, KCl, K_2_SO_4_, Na_2_SO_4_, glucose, glutamate, glycine and lysine.

## 4. Conclusions

In this work, Ti_3_C_2_T*_x_* coated with TiO_2_ nanosheets was synthesized using a simple solvothermal process. The Ti_3_C_2_T*_x_*@TiO_2_ NSs-modified GCE has been proven to enable the simultaneous detection of AA at concentrations ranging from 200 to 1000 μM, DA at concentrations ranging from 40 to 300 μM, and UA at concentrations ranging from 50 to 400 μM. The LOD achieved for these analytes is 2.91 μM (AA), 0.19 μM (DA) and 0.25 μM (UA). Furthermore, the Ti_3_C_2_T*_x_*@TiO_2_/GCE exhibits excellent resistance to interference, good repeatability and high selectivity. Two key advantages of the Ti_3_C_2_T*_x_*@TiO_2_ are demonstrated to enhance their capability for simultaneous detection. Firstly, the substitution of –F ions with additional Ti^3+^ ions leading to a negative to neutral surface charge change of Ti_3_C_2_T*_x_*, thereby reducing electrostatic repulsion. Secondly, the growth of a mass of TiO_2_ nanosheets on the surface of Ti_3_C_2_T*_x_* significantly increases the specific surface area and charge transport properties of the composite material. This work provides a novel approach for AA, DA and UA recognition in MXene-based composites. In future electrochemical detection, researchers may utilize targeted nanomaterial modifications between MXene lamellae to achieve precise detection of various analytes. This approach not only mitigates the self-accumulation of MXene but also increase the specific active sites, which is conducive to improving the selectivity and sensitivity of MXene-based materials.

## Data Availability

Date will be made available on request.

## Supplementary Materials

The following supporting information can be downloaded at: https://www.mdpi.com/article/10.3390/molecules29122915/s1. Figure S1. XRD patterns of Ti_3_AlC_2_, Ti_3_C_2_T*_x_* and Ti_3_C_2_T*_x_*@TiO_2_ NSs. Figure S2. SEM images of (a) Ti_3_AlC_2_, (b)Ti_3_C_2_T*_x_.* Figure S3. (a) DPV results in the presence of 1 mM AA at different concentrations of DA (80–500 μM) (b) the relationship between the corresponding oxidation peak current value and concentration of DA (c) DPV results in the presence of 20 μM DA at different concentrations of AA (150–400 μM), (d) the relationship between the corresponding oxidation peak current value and concentration of AA. Figure S4. (a) DPV results in the presence of 150 μM UA at different concentrations of DA (50–400 μM) (b) the relationship between the corresponding oxidation peak current value and concentration of DA (c) DPV results in the presence of 100 μM DA at different concentrations of UA (150–400 μM), (d) the relationship between the corresponding oxidation peak current value and concentration of DA. Figure S5. (a) DPV results in the presence of different concentrations of AA (200–700 μM) and UA (100–350 μM), (b) DPV results in the presence of 500 μM AA at different concentrations of UA (40–100 μM), (c) DPV results in the presence of 100 μM UA at different concentrations of AA (40–500 μM).
