# Supplementary material for: Ti3C2Tx Coated with TiO2 Nanosheets for the Simultaneous Detection of Ascorbic Acid, Dopamine and Uric Acid
Source: Molecules. 2024 Jun 19;29(12):2915. doi: 10.3390/molecules29122915 (PMC11206739; doi:10.3390/molecules29122915)
Supplement: Supplementary file 1 [file molecules-29-02915-s001.zip › molecules-3016517-supplementary.pdf]

Supplementary Information

$Ti_3C_2T_x$ Coated with TiO_2 Nanosheets for the Simultaneous Detection of Ascorbic acid, Dopamine and Uric Acid

Dengzhou Jia [1], Tao Yang [1,2,*], Kang Wang [1], Hongyang Wang [3,*], Enhui Wang [1,2], Kuo-Chih Chou [1] and Xinmei Hou [1,2,4,*]

[1] Institute for Carbon Neutrality, University of Science and Technology Beijing, Beijing 100083, China
[2] Institute of Steel Sustainable Technology, Liaoning Academy of Materials, Shenyang 110167, China
[3] State Key Laboratory of Environmental Criteria and Risk Assessment, Chinese Research Academy of Environmental Sciences, Beijing 100012, China
[4] Beijing Advanced Innovation Center for Materials Genome Engineering, University of Science and Technology Beijing, Beijing 100083, China
* Correspondence: yangtaoustb@ustb.edu.cn (T.Y.); wanghongyang_why@126.com (H.W.); houxinmeiustb@ustb.edu.cn (X.H.)

1. Characterization of Ti_3AlC_2, $Ti_3C_2T_x$ and $Ti_3C_2T_x$@TiO_2 NSs

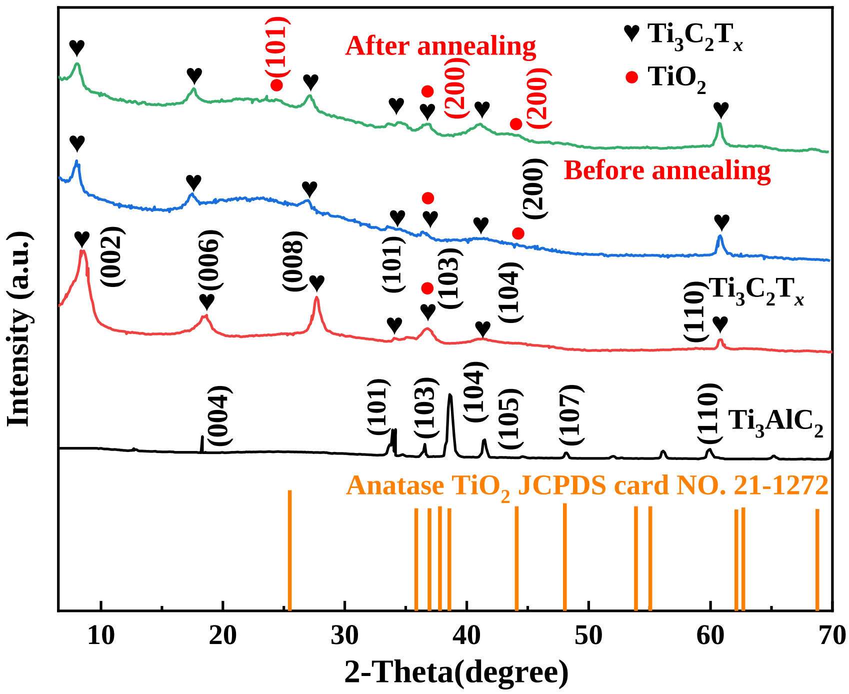

Figure S1. XRD patterns of Ti_3AlC_2, $Ti_3C_2T_x$ and $Ti_3C_2T_x$@TiO_2 NSs.

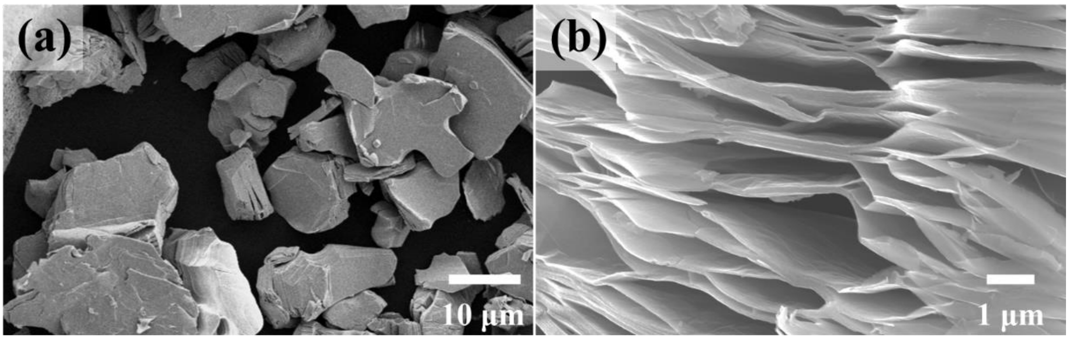

Figure S2. SEM images of (**a**) Ti_3AlC_2, (**b**)$Ti_3C_2T_x$.

2. Simultaneous measurement of AA, DA, and UA

The DPV process was undertaken to assess the efficacy of electrodes modified with $Ti_3C_2T_x/TiO_2$ NSs. Figure S3 presents the DPV curves acquired at varying concentrations for AA and DA. As depicted in Figure S3a, the experimental investigation focused on fluctuating the concentration of DA (50–400 μM), while maintaining constant concentrations of AA (1000 μM). The oxidation peak currents exhibited an increase with the introduction of DA. Conversely, the oxidation peak currents of AA showed no significant rise and did not interfere with DA. Similarly, an anti-interference test was conducted involving AA concentrations ranging from 150–400 μM in the presence of 20 μM DA (Figure S3c). This observation indicated the distinct presence of oxidative peaks for both AA and DA at 0.23 and 0.45 V, respectively, according to the DPV results. The DPVs demonstrated good linear responses for AA (R^2 = 0.9917) and DA (R^2 = 0.9871) (Figure S3b, d).

Figure S4 illustrates the DPV curves obtained at different concentrations for DA and uric acid (UA). As shown in Figure S4a, the experimental setup involved fluctuating the concentration of DA (50–400 μM), with UA maintained at a constant concentration of 150 μM. The oxidation peak currents increased upon the addition of DA. Simultaneously, the oxidation peak currents of UA did not exhibit significant escalation and did not interfere with DA. Similarly, an anti-interference study was conducted involving UA concentrations ranging from 150–400 μM in the presence of 100 μM DA (Figure S4c). This highlighted the presence of well-defined oxidative peaks for both DA and UA at 0.30 and 0.45 V, according to the DPV results. The DPVs displayed good linear responses for DA (R^2 = 0.9933) and UA (R^2 = 0.9985) (Figure S4b, d).

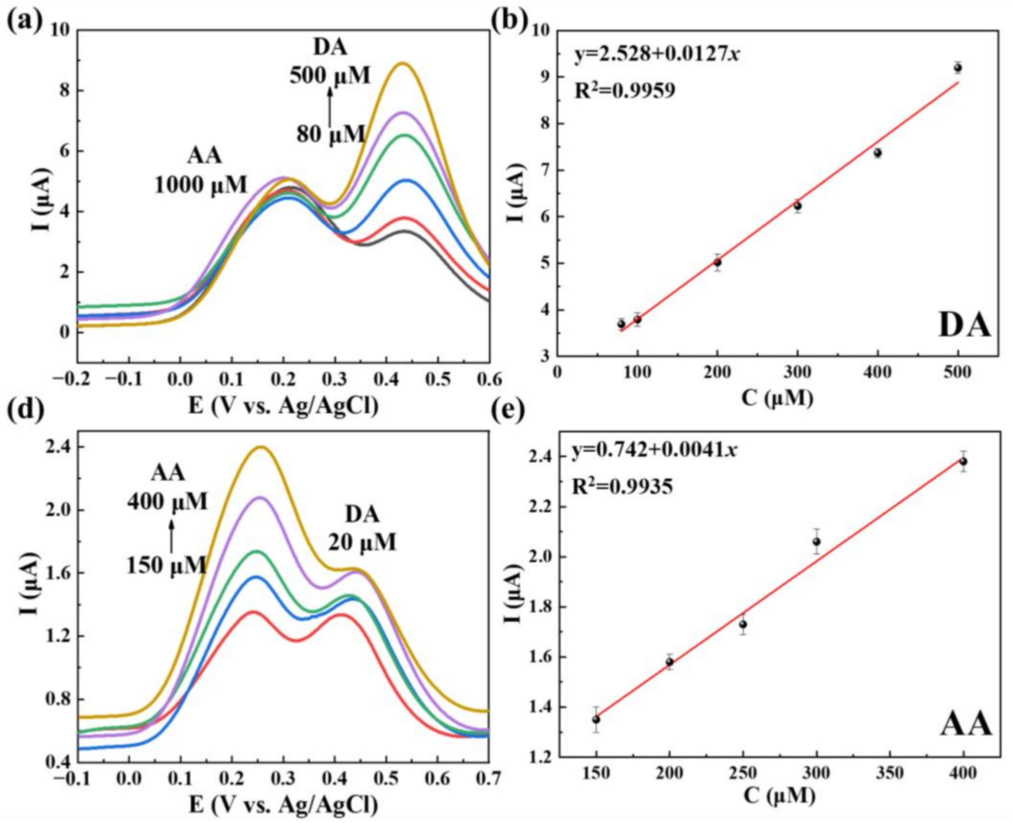

Figure S3. (**a**) DPV results in the presence of 1 mM AA at different concentrations of DA (80–500 μM) (**b**) the relationship between the corresponding oxidation peak current value and concentration of DA (**c**) DPV results in the presence of 20 μM DA at different concentrations of AA (150–400 μM), (**d**) the relationship between the corresponding oxidation peak current value and concentration of AA.

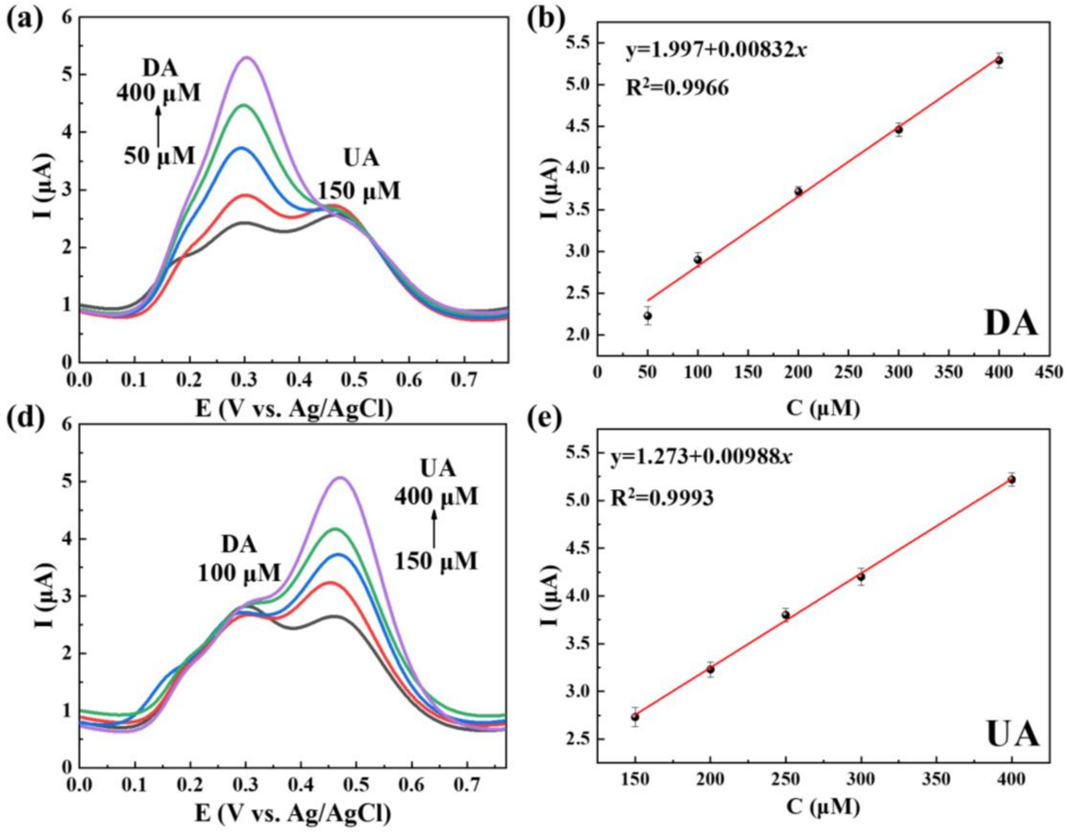

Figure S4. (**a**) DPV results in the presence of 150 μM UA at different concentrations of DA (50–400 μM) (**b**) the relationship between the corresponding oxidation peak current value and concentration of DA (**c**) DPV results in the presence of 100 μM DA at different concentrations of UA (150–400 μM), (**d**) the relationship between the corresponding oxidation peak current value and concentration of DA.

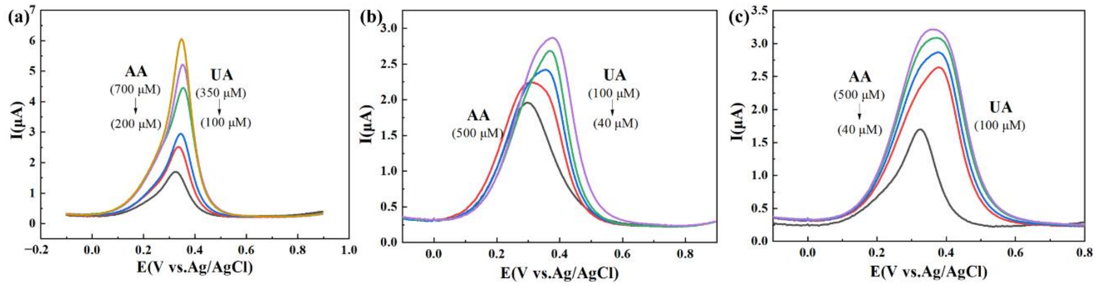

Figure S5. (**a**) DPV results in the presence of different concentrations of AA (200–700 μM) and UA (100–350 μM), (**b**) DPV results in the presence of 500 μM AA at different concentrations of UA (40–100 μM), (**c**) DPV results in the presence of 100 μM UA at different concentrations of AA (40–500 μM).

3. Illustration of the equation for the formation of TiO_2 NSs

$$Ti^{3+} + H_2O \rightarrow TiOH^{2+} + H^+ \quad (S1)$$

$$TiOH^{2+} + O^2 \rightarrow Ti(\text{IV})\ \text{oxo species} + O^{2-} \quad (S2)$$

$$Ti^{4+} + HO - CH_2 - CH_2 - OH \rightarrow Ti(OCH_2CH_2O)_2 + 2H^+ \quad (S3)$$

$$Ti(OCH_2CH_2O)_2 \rightarrow TiO_2\ (\text{Solvothermal,} \quad \text{Annealing}) \quad (S4)$$
